# Imaging clinically relevant pain states using arterial spin labeling

**DOI:** 10.1097/PR9.0000000000000750

**Published:** 2019-08-07

**Authors:** Marco Luciano Loggia, Andrew Reilly Segerdahl, Matthew Alexander Howard, Irene Tracey

**Affiliations:** a Department of Radiology, A. A. Martinos Center for Biomedical Imaging, Harvard Medical School, Massachusetts General Hospital, Boston, MA, USA; b Nuffield Department of Clinical Neuroscience, Wellcome Centre for Integrative Neuroimaging, FMRIB, University of Oxford, Oxford, United Kingdom; c Department of Neuroimaging, Institute of Psychiatry, Psychology and Neuroscience, King's College London, London, United Kingdom

**Keywords:** Arterial spin labeling, Perfusion, Regional cerebral blood flow, Pain, Humans, Tonic, Clinical

## Abstract

Arterial spin labeling (ASL) is a perfusion-based functional magnetic resonance imaging technique that uses water in arterial blood as a freely diffusible tracer to measure regional cerebral blood flow noninvasively. To date, its application to the study of pain has been relatively limited. Yet, ASL possesses key features that make it uniquely positioned to study pain in certain paradigms. For instance, ASL is sensitive to very slowly fluctuating brain signals (in the order of minutes or longer). This characteristic makes ASL particularly suitable for the evaluation of brain mechanisms of tonic experimental, postsurgical, and ongoing/or continuously varying pain in chronic or acute pain conditions (whereas blood-oxygen level–dependent functional magnetic resonance is better suited to detect brain responses to short-lasting or phasic/evoked pain). Unlike positron emission tomography or other perfusion techniques, ASL allows the estimation of regional cerebral blood flow without requiring the administration of radioligands or contrast agents. Thus, ASL is well suited for within-subject longitudinal designs (eg, to study evolution of pain states over time, or of treatment effects in clinical trials). Arterial spin labeling is also highly versatile, allowing for novel paradigms exploring a flexible array of pain states, plus it can be used to simultaneously estimate not only pain-related alterations in perfusion but also functional connectivity. In conclusion, ASL can be successfully applied in pain paradigms that would be either challenging or impossible to implement using other techniques. Particularly when used in concert with other neuroimaging techniques, ASL can be a powerful tool in the pain imager's toolbox.

## 1. Introduction

In the last 25 years, functional brain imaging has been used to gain unprecedented access to the neural mechanisms underlying many mental, perceptual, and sensory processes, including the experience of pain. Through the use of techniques such as functional magnetic resonance (fMRI), electroencephalography and magnetoencephalography (EEG and MEG, respectively), and positron emission tomography (PET), we have, for example, discovered that multiple brain networks are involved in different aspects of pain processing, confirmed in humans the existence of a descending pain modulatory system (DPMS) and its involvement in pharmacological and nonpharmacological analgesia, and identified pathological alterations in the brains of chronic pain patients.^3,61,76,89^

Despite the undeniable wealth of information that these commonly used techniques have generated, each of them naturally demonstrates a profile of strengths and weaknesses which renders them best-suited to capture the brain activity associated with certain types of pain, or certain aspects of the pain experience, but not others. For instance, blood-oxygen level–dependent (BOLD) fMRI is very sensitive to fluctuating brain signals of the order of seconds, but comparatively insensitive to those at lower frequencies (ie, minutes, hours, etc).^1^ Because of this characteristic, BOLD fMRI has been particularly successful in the identification of brain activity in response to evoked/phasic pain—arguably more relevant to short-lasting acute pain conditions, whereas the brain activations/deactivations induced by chronic pain (typically evolving over much longer time scales; from minutes to hours, weeks, months, or years) have largely remained elusive. Certainly, many laboratories around the world have used BOLD fMRI to investigate the neural mechanisms of different types of clinical pain (whether acute or chronic) by either performing functional connectivity analyses to identify changes in the way brain regions or networks communicate with each other in patients with pain disorders, or evoking clinical symptoms and examining the neural response (eg, allodynia, hyperalgesia, and movement-related pain). However, only a few examples of BOLD fMRI studies evaluating ongoing/slowly varying or constant pain can be found in the literature.^4^ In principle, PET could be used to investigate brain changes evolving over minutes,^11,12,20,36,42,73,75,84,86,88,94^ but its spatiotemporal resolution, invasiveness, hardware demands (eg, the need for an on-site cyclotron if using radioligands with a short half-life), relative inflexible paradigm design opportunities, cost, and the use of radiation limit its application.

In the last 10 years, neuroscientists studying pain have begun adding a tool—previously used in other areas of sensory and perceptual neuroscience as well as stroke research—to their toolbox: arterial spin labeling (ASL). Arterial spin labeling is a perfusion-based fMRI technique which uses water in arterial blood as a freely diffusible tracer to measure regional cerebral blood flow (rCBF) noninvasively. Several features of ASL detailed here, namely, its sensitivity to low-frequency signals, its ability to generate data that can be quantified in absolute units (mL blood/100 g tissue/min) while not requiring an intravenous administration of a radiopharmaceutical or contrast agent, make it uniquely positioned to offer novel insights on brain mechanisms of pain, especially when combined with other powerful and commonly used neuroimaging techniques.

## 2. How does arterial spin labeling work?

The basic principle of ASL is that perfusion can be measured noninvasively by converting arterial blood flowing to the brain into a “magnetic tracer.” When the brain is perfused with magnetically labeled blood, its MRI signal becomes slightly (∼0.5–1.5%) reduced in a manner that is proportional to the amount of magnetic “label” that has flowed there. By quantifying signal loss induced by the labeling, we can calculate rCBF throughout the brain. This process is safe, noninvasive, and does not involve the injection of a contrast agent.

**Figure 1.**
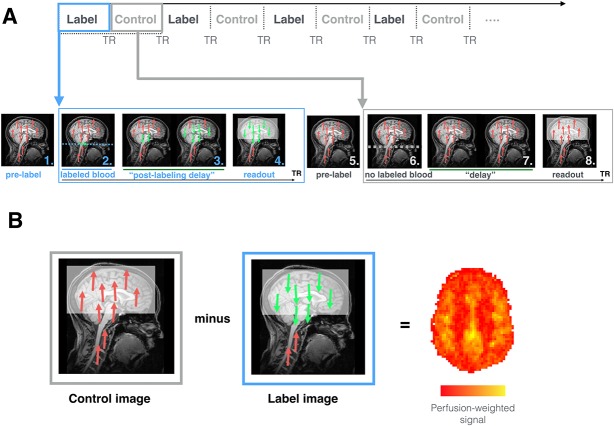
How ASL works. (A) Sequence of events occurring during ASL image acquisition. During labeling (blue), the following steps occur. 1. As the subject is placed in the MRI scanner and in the absence of any MR pulses, the body's protons, including those in blood-water (red), are aligned with the main magnetic field. 2. Blood–water (red) flowing through the labeling region (in this case a plane; blue dotted line), is rotated out of phase with the main magnetic field. Labeled blood is displayed in green. 3. The “postlabeling delay” is equivalent to a “waiting period” wherein labeled blood (green) is able to flow from the labeling region to brain tissue before the images are collected. 4. Images are acquired that capture the magnitude of labeled blood (green) that has arrived at the tissue. During the acquisition of control images (gray), the same sequence of steps occurs except no blood is labeled. 5. Blood–water (red) is aligned with the main magnetic field. 6. Blood–water (red) flowing through the labeling region (dotted line) are NOT rotated out of phase with the main magnetic field. This means that the blood water is not magnetically labeled. 7. A waiting period identical to the “postlabeling delay” is allowed to elapse, except that here the blood is not labeled (red). 8. Images are acquired that capture the magnitude of background “static” tissue signal where no blood is labeled. (B) During postprocessing, the subtraction between “control” and “label” volumes allows for the calculation of perfusion-weighted maps. The later can be further processed to obtain an rCBF map that is fully quantitative (mL blood/100 g tissue/min). ASL, arterial spin labeling; rCBF, regional cerebral blood flow; TR, repetition time.

Arterial spin labeling sequences have fundamental principles in common. These involve labeling arterial blood upstream from the imaging volume in the brain, waiting for the labeled blood to flow to the imaging volume (often referred to as the “postlabeling delay”), and then acquiring images. Immediately after this, a “control” image is also collected in which no label is applied (ie, arterial blood is not magnetically labeled). By computing the difference between “control” and “label” images, perfusion can be calculated using a standard ASL analysis pipeline (Fig. 1). A description of each step in this process follows. For a full account of the physics behind the ASL signal, please see Refs. 21, 70, 74, and 93.

### 2.1. Labeling

Labeling (or “tagging”) is the process of magnetically inverting or saturating the hydrogen nuclei (also known as “spins”) in water molecules stored within arterial blood as it passes through a labeling region. In general, labeling is made possible by the application of radiofrequency (RF) energy applied to a specific spatial location upstream with respect to the arterial blood flow (eg, at the base of the brain/in the neck). Different types of ASL sequences are focused on optimizing how the arterial blood is labeled and for how long—key variables necessary to bolster the signal-to-noise ratio (SNR) of the acquired images (for review^13^). For instance, depending on the ASL approach used, labeling can be achieved by continuously labeling blood (continuous ASL [cASL]), using a single pulse (pulsed ASL [pASL]) or a train of discrete radiofrequency pulses to mimic continuous labeling (pseudocontinuous ASL [pcASL]). Of the 3 sequence types, cASL typically achieves the highest SNR, but requires an external RF coil, is difficult to use with whole-brain multislice acquisitions, has specific absorption rate limitations, and is poorly suited for use on most clinical scanners where the amplifiers are typically incapable of meeting the energy demands of cASL sequences.^19^ PASL does not necessitate additional hardware but achieves the lowest SNR. Pseudocontinuous ASL offers the best of both worlds by achieving improved SNR without imposing additional hardware requirements, which is among the reasons why pcASL is the recommended approach, as defined by the ASL consensus paper.^2^

Generally, sequences that use a longer label duration will ensure more labeled blood is available to perfuse brain tissue, resulting in a larger ASL signal. This relationship is maintained as long as the labeling duration is not much longer than the relaxation time of blood, (ie, ∼1.6 seconds at 3T^2,56^). However, longer durations may be difficult to obtain for technical or safety reasons (eg, excessive power deposition) and require longer repetition times (TRs—see below), which ultimately lead to a lower number of averages obtained within a given scan.^2^ A label duration of 1.8 seconds is generally believed to represent an acceptable compromise solution.^2^

Of course, achieving proper labeling is paramount in ASL. This process can be compromised by a suboptimal placement of the labeling region and/or the presence of magnetic inhomogeneities. For maximum labeling efficiency, the labeling region should be perpendicular to the major ascending arteries. If the labeling plane is poorly aligned with the participant's vasculature, adiabatic inversion of the blood–water protons will be suboptimal, reducing SNR. One solution is to visualize the participant's vasculature using a “time of flight” angiography scan before the ASL scans are run. The experimenter can use this to guide the placement of the labeling plane where there are no occlusions due to kinking of vessels.

### 2.2. Postlabel delay time

Once labeled, the hydrogen nuclei within blood water are thermodynamically unstable, which results in a slight reduction in their MR signal—a feature that can be exploited to generate functional images. These images are collected after a brief period (typically referred to as a “delay time” or “postlabeling delay” [PLD]), in which the labeled arterial blood is able to flow from the labeling plane (or slab) to the brain tissue (Fig. 1A).

The primary factor guiding the selection of a PLD time is that this needs to be long enough to allow the labeled arterial blood to transit from the labeling region to capillaries within the imaging volume (ie, arterial transit time [ATT]). However, if the PLD becomes too long, the label is no longer observable because of the fact that the hydrogen nuclei gradually relax back into phase with the magnetic field.^2^ The consensus paper states that a PLD time of 1.8 seconds is generally appropriate for healthy human adults younger than 70 years, whereas it should be increased to 2 seconds for older participants and some clinical populations.^2^ In general, single-delay ASL approaches will give accurate rCBF measurements as long as the ATTs across the brain are shorter than the delay time. However, with single-delay approaches, there is no way to know whether this is the case with absolute certainty because there is no way to accurately measure ATT. One solution to this problem is to use a delay time that is very long to ensure a net maximum of arterial signal arrives at all voxels in the brain. However, as previously mentioned, this comes at a cost of SNR due to T1 relaxation of the arterial signal because the magnetic label will be lost once the arterial spins relax back into alignment with the scanner's magnetic field.

### 2.3. Control images

Because the hydrogen nuclei in water are not restricted to arterial flow—but exist in all brain tissue—it is essential to collect control images in which no arterial blood is labeled, to remove the contribution of static tissue-derived signals to the label images. To do this, all aspects of the ASL sequence protocol remain the same except that the arterial blood is not labeled (eg, the RF energy is not applied to the labeling region, or it is applied but off-resonance, thereby resulting in an ineffective label) (Fig. 1B). Each pair of “label” and “control” images can then be subtracted to generate a perfusion-weighted image, which is an estimate of how much arterial labeled blood has perfused the brain tissue. The perfusion-weighted signal is higher for active regions because their higher demand for oxygen leads to larger amounts of labeled blood reaching them, causing a larger difference between control and label images. This difference is usually very small (approximately 1%) and is a primary reason why ASL has an inherently low SNR. A number of sequence parameter optimizations exist to mitigate this limitation (for review see^13^). Primarily, however, it is essential to collect as many pairs of “label” and “control” images as is permissible with the particular experimental paradigm being utilized.

### 2.4. Quantification

The difference between each “control-label” pair of images reveals the perfusion-weighted signal at each voxel (Fig. 1B). However, the intensity values observed here are not yet in physiological units. To quantify rCBF, 2 additional steps must occur: the data must be fitted to a kinetic model and then calibrated.

All functional imaging methods use models that relate neural activity to changes in perfusion (ie, neurovascular coupling) to interpret the images collected in a physiologically meaningful way. For example, BOLD fMRI uses the haemodynamic response function (HRF) as a model of changes in the ratio of deoxygenated to oxygenated blood when neurons are active. BOLD fMRI paradigms will sample the HRF once per “repetition time” (TR; ie, the time between each consecutive volume acquisition). To ensure that the HRF is sampled accurately, it is common practice to vary the timing of the TR relative to the stimulus presentation (ie, “jittering”). This ensures that the shape of the HRF is accurately sampled given the paradigm being tested.

Similarly, ASL uses the kinetic model to describe the relationship between the arterial signal magnitude and perfusion.^9^ This is an equation that describes the dynamics of how the labeled blood arrived (ATT) and how the magnetization signal changes over time within a voxel—features that are related to key variables such as the rate of arterial delivery, blood equilibrium magnetization, labeling duration, and T1 relaxation times.

**Figure 2.**
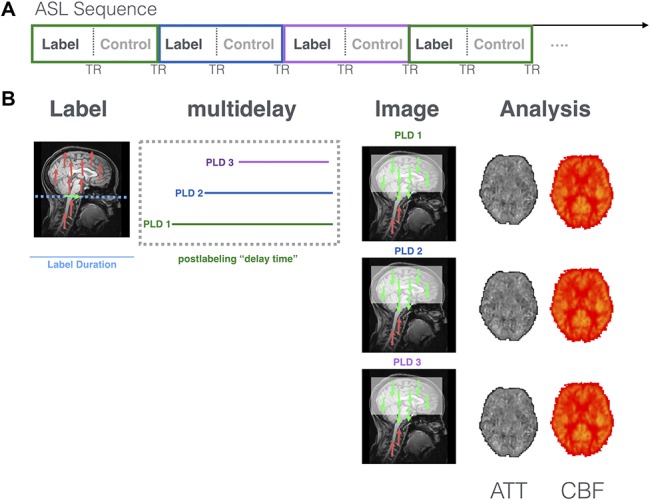
How multidelay ASL works. Multidelay ASL sequences use a range of different PLD times, which are preselected based on the protocol used. (A) Each PLD is represented in the figure by a colored line (eg, green, blue, and purple) that represents how long the scanner waits before the brain image is collected. The same PLD is used for each “control” and “label” pair of images. (B) Postprocessing of multidelay data allows for the calculation of both the time at which the labeled blood first arrives (ie, ATT: arterial transit time) and rCBF (mL blood/100 g tissue/min) at each voxel. ASL, arterial spin labeling; ATT, arterial transit time; PLD, postlabeling delay; rCBF, regional cerebral blood flow; TR, repetition time.

The common principle here is that both the HRF and the kinetic model are equations that describe unique features of neurovascular coupling physiology. In the case of single-delay methods, the kinetic curve is sampled once per TR and then perfusion-weighted images are averaged over time to boost SNR. An alternative approach is to acquire perfusion-weighted images using a range of delay times optimized to capture both the ATT and rCBF across all voxels in the brain in order for the kinetic curve to be sampled in its entirety.^9,24,27,60^ Postlabeling delays of varying lengths are used, cycled through sequentially (one per TR) over the course of the acquisition (Fig. 2A). These data can then be fitted using a kinetic model without the assumption that all labeled blood has perfused the voxel at a given delay time. By sampling magnetization intensity at multiple delay times (ie, multidelay, or multi-PLD, ASL), it is possible to reliably calculate both the time at which the arterial signal first arrives (ATT) and a measure of the arterial magnetization at the peak in each voxel (Fig. 2B). So, even if these variables vary gradually over the course of the paradigm, either due to a change in the pain experienced (or say, the application of an anesthetic), the multidelay method is still sensitive to these changes and, as such, provides a more accurate calculation of blood perfusion.

The advantage of multi-PLD ASL is particularly relevant when one wants to know whether a particular brain region is more active than another distant region—ie, whether this activity difference has possible biological relevance. By sampling the full kinetic curve, using a multidelay approach, we be more certain that a difference in perfusion is meaningful rather than due to sampling error.

One downside of using multiple delays is that the more PLDs are used, the longer the scan duration. An increase in scan duration might sometimes not be possible or advisable, particularly in experiments where saving scan time is crucial (eg, when scanning chronic pain patients suffering a pain attack in the scanner). However, if the experimental design permits it and there is time to scan, the multidelay method can provide more accurate and precise quantification of absolute perfusion compared with single-delay methods.^13,28,98^

Once the model fitting step is complete, the last step is to calibrate the data. Here, the perfusion images are scaled based on the total concentration of arterial signal that is available for labeling in a participant. To do this, typically a proton density–weighted image is collected (ie, a calibration, or M_0_, image), which measures the magnetization of hydrogen nuclei in water in both tissue and blood. This will vary between subjects (and scanners), so it needs to be calculated for each subject's scan. The end product of these steps is a perfusion image quantified in absolute physiological units (mL blood/100 g tissue/min).

The possibility of quantifying rCBF in physiological units is an important difference between ASL and BOLD fMRI. Because the BOLD FMRI signal is susceptible to nonphysiological factors (eg, gradient heating, time of day, scanner type, and site), its absolute units are physiologically meaningless, which is why fMRI studies detect changes in brain activation by evaluating relative differences (eg, between epochs of an active task and rest periods). By contrast, when ASL images are calibrated, nonphysiological sources of variability in the signal are accounted for, allowing for the full quantification of the signal in absolute, physiological units. An absolute signal means that ASL data can be more reliably compared across different settings and scanners, and the effects can be interpreted as reflecting more direct relationships between the experimental conditions and the underlying physiology evaluated.

### 2.5. Application

Despite its powerful features, ASL has largely remained a “niche technique” for several years after its invention in the nineties.^21^ The reasons for this are varied and are likely to include early issues with poor sensitivity, a lack of clarity about which ASL method is best to use, limited access from scanner manufacturers to specific ASL sequences themselves, and the availability of analysis packages needed to quantify rCBF. Their use was restricted to laboratories with MR physicists specializing in the method, and most applications focused on measuring perfusion in stroke patients. Moreover, although extensive, the literature on ASL can be highly technical, and often it can be difficult to ascertain the feasibility (or benefit) of translating these advances into a clinical research setting: it simply can be challenging to determine which “flavor” of ASL is optimal for different experimental contexts. More recently, however, ASL has begun to be adopted more widely by the neuroimaging community thanks to recent technical improvements (such as the development of higher field strength magnets and phased array receiver coils), the development of software packages for data processing and analysis, such as ASLtbx (https://cfn.upenn.edu/∼zewang/ASLtbx.php) and BASIL from the FSL suite (https://fsl.fmrib.ox.ac.uk/fsl/fslwiki/BASIL), and the fact that ASL sequences are now offered as a product by all major scanner manufacturers. Furthermore, the community has begun producing consensus papers, a testament to the growing number of users, such as that by the ISMRM Perfusion Study Group and the European Consortium for ASL in Dementia, providing specific recommendations for a standard approach.^2^ Nonetheless, although applications have moved beyond stroke, they still largely focus on changes in resting perfusion as an indicator of disease (eg, CBF compromises) rather than as an indicator of changes in neural activity during a sensory stimulation or cognitive process—akin to our use of BOLD imaging in other areas of imaging neuroscience. In fact, there is no conceptual reason why ASL could not be used for paradigms with both short and long duration events—with several advantages. Although BOLD contrast is undoubtedly the most commonly used neuroimaging method for studies of brain activity, the underlying physiological processes giving rise to measured BOLD signal changes (which include contribution from changes in CBF, cerebral blood volume, and cerebral metabolic rate of oxygen consumption [CMRO_2_]) vary substantially between sessions and individuals. Therefore, there might be a superiority of ASL over BOLD in terms of location of activation (less biased by larger blood vessels known as “draining veins”) and reproducibility across sessions—one early study by Tjandraet al.^87^ assessed this question of superiority by comparing the localization of activation and reproducibility of relative signal change measured by optimized BOLD vs ASL-derived CBF. Data were collected within the primary sensorimotor cortex in normal healthy controls performing a simple finger-tapping task over 3 imaging sessions (2 on same day and 1 on a different day). The displacement between the foci of BOLD and CBF activation was less than the linear dimension of one voxel (2.4 mm); however, BOLD activation was significantly closer to the nearest draining vein compared with CBF activation. For the relative signal change measurement, CBF had a significantly lower intersubject variation compared with BOLD, enabling a smaller sample size for any given effect size, although the intrasubject variation across sessions for CBF was not significantly different from BOLD.

Despite the advantages of ASL, for reasons given above regarding access, ease of use, and analysis, BOLD still dominates the neuroimaging space when it comes to event-related or shorter-lasting changes in neural activity. However, the field of ASL is developing rapidly with a growing interest from neuroscientists seeking to use it for novel paradigm applications to which the BOLD method is poorly suited (eg, the study of tonic itch states^37,69^). Recognition that contemporary ASL techniques offer a powerful means of interrogating a range of pathophysiological features in both human and nonhuman species, across a large swathe of both clinical and related experimental domains is gathering pace—of which pain is exceptionally well-suited to benefit.

## 3. Imaging acute pain with arterial spin labeling

**Figure 3.**
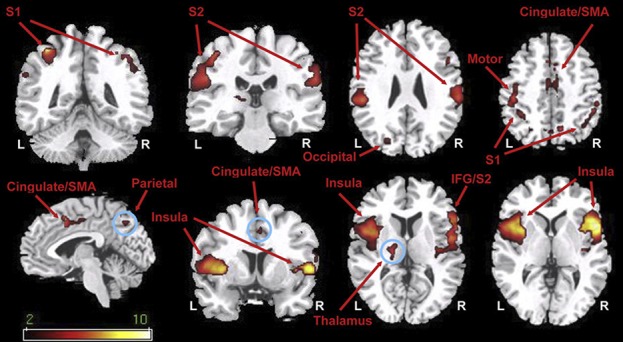
Imaging acute experimental pain with ASL. Elevations in rCBF in response to 60 seconds of contact heat pain stimuli compared with rest (ie, no pain) (from Ref. 65). ASL, arterial spin labeling; IFG, inferior frontal gyrus; L, left side; R, right side; S1, primary somatosensory cortex; S2, secondary somatosensory cortex; rCBF, regional cerebral blood flow; SMA, supplementary motor area.

Only as recently as in 2008 have neuroscientists begun to apply ASL to the study of pain. In the first study, Owen et al. have demonstrated that the stimulation of the left hand with 1-minute heat pain stimuli in healthy volunteers was associated with rCBF increases of the order of 3 to 5 mL/100 g/min in multiple regions including insula, primary (S1) and secondary (S2) somatosensory cortices, thalamus, cingulate cortices, and supplementary motor area^65^ (Fig. 3). After this seminal paper, other laboratories have investigated the brain processing of acute pain using ASL.^25,58^ In a study by Maleki et al., for instance, 15-second contact heat stimuli on the dorsum of the hand induced responses in regions such as insula, thalamus, hippocampus, amygdala, anterior cingulate, primary and secondary somatosensory cortex, precentral gyrus, and precuneus.

Overall, these studies show that pain-related rCBF changes can be observed in brain regions commonly described as activated by acute pain stimuli in imaging studies using fMRI, PET, or other techniques,^3^ validating the utility of ASL as a tool to image brain responses to some types of acute pain.^58^

Although ASL can be successfully adopted to investigate brain responses to acute pain, it should also be noted that the temporal resolution of ASL is typically lower than in BOLD fMRI. As each perfusion-weighted volume is the result of the subtraction between control and label volumes, its “effective TR” is doubled (ie, the TR of the control volume plus that of the label volume). Because the TR for a single ASL volume is itself slightly longer (often ∼3–5, vs ∼2 seconds in a typical whole-brain BOLD acquisition), this results in a significantly lower sampling rate compared with BOLD fMRI. This characteristic, paired to the intrinsically lower SNR, generally might make ASL a less-sensitive technique to detect brain activations/deactivation in response to acute pain stimuli,^90^ particularly in event-related designs (ie, in which the stimuli are presented for very brief periods of time). Of course, this drawback can be obviated by lengthening the duration of the scan and allowing for a higher number of stimulus presentations. Moreover, even in the context of acute pain, ASL retains the advantage of being fully quantitative and yielding rCBF estimates in absolute, physiological units.

## 4. Imaging experimental tonic and ongoing pain with arterial spin labeling

We have gained vast knowledge about the brain mechanisms underlying the experience of acute pain by using BOLD fMRI. However, it is not yet well understood the extent to which the regions identified in acute paradigms remain active and engaged during pain experiences that extend for long periods and whether these regions are specific to pain or more accurately reflect the multidimensional elements of the pain experience and, as a consequence, are multimodal (ie,“domain-general” emotional, cognitive, and sensory processing regions). Furthermore, it must be stated that the sensation evoked by an acute cutaneous input is not equivalent to the core pain features that patients are typically seeking treatment for—namely, pain that is deep, ongoing, and spontaneously fluctuating. Implementation of imaging approaches that are able to capture these approaches are essential to understand the core mechanisms that promote and maintain different pathological pain states.

Two important studies, by Owen et al.,^66,67^ provided strong evidence that ASL could be used effectively to this end. In these studies, the authors adapted an established tonic muscle pain paradigm^10,75,83,86^ to an ASL setting. In their 2010 study, the authors injected healthy participants with an initial bolus followed by a continuous infusion of hypertonic saline (5% NaCl) into the brachioradialis muscle for 15 minutes while being scanned. The primary aim of this study was to observe which brain regions were activated by the initial bolus (ie, acute pain onset) and remained activated and engaged for the duration of the continuous 15-minute infusion. This study showed that while a number of regions were active acutely (eg, cingulate, insula, and thalamus), only the insular cortex remained engaged throughout the course of the infusion and was the region that demonstrated the strongest positive correlation between rCBF increases and participants' self-reports of the severity of the pain experienced.^67^

A follow-up investigation by Owen et al.^66^ aimed to replicate these data in a new cohort that experienced only the slow, ongoing infusion of hypertonic saline (ie, no acute bolus injection). Data from this study confirmed that acute and tonic phases of pain are linked to different stimulus response profiles in active cortical regions, and the only region recruited during both the acute and tonic phases of the paradigm was the bilateral anterior insula cortex. However, unlike their previous study in 2010, these data did not show a strong coupling between CBF changes and the pain intensity reported during the slow infusion paradigm—a result which the authors suggested could be related to a combination of physiological, psychological, and methodological factors.

These data are intriguing for a number of reasons. Primarily, they provide evidence of nonuniformity in the stimulus response profiles of brain regions activated by pain experiences that extend for more than a few minutes and entail dynamic changes in intensity. Previously, key work by Coghill et al.^17^ used ^15^O water PET to identify a number of brain regions capable of tracking graded changes in cutaneous heat applied to participants' arm. Here, the primary finding was that rCBF changes were positively correlated with the stimulus intensity in regions such as the primary and secondary somatosensory cortices, insula, anterior cingulate cortex, putamen, thalamus, and cerebellum. Consistent with this observation, the Owen et al. 2010 study,^67^ which involved an acute bolus injection of hypertonic saline, showed a strong positive relationship between the initial peak pain intensity of the saline infusion and rCBF, in regions such as the anterior midcingulate, insula, and thalamus, for a period analogous to the duration of the heat stimuli used by Coghill et al. (eg, 2.5 minutes). However, as the stimulus extended for longer periods, most of these regions did not remain activated and engaged on a timescale that aligns with the dynamics of the stimulus. Although a number of factors are likely to underlie this observation including habituation, fundamental differences in the nociceptor subtypes engaged, the afferent pathways that are activated (eg, superficial vs deep laminae), and the pain qualia experienced (deep muscle ache vs oscillating topical heat), these data suggest that the encoding of ongoing pain is likely to vary in a highly dynamic way that changes nonlinearly in a stimulus duration–dependent manner throughout the entire pain neuraxis.

**Figure 4.**
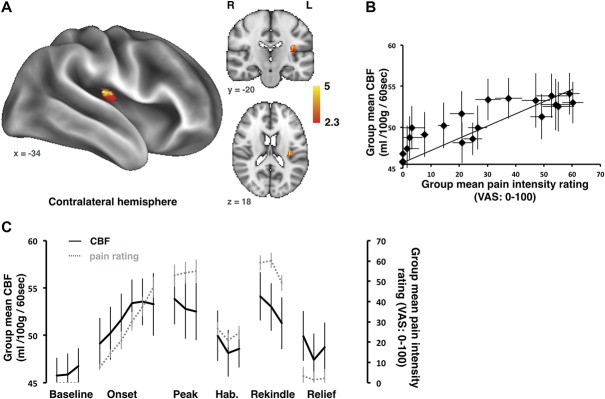
Imaging ongoing experimental pain with ASL. Whole-brain absolute CBF correlation with pain intensity ratings during an ongoing pain experience induced by topical capsaicin and thermal stimulation. (A) The contralateral dorsal posterior insula (dpIns) showed a strong correlation between absolute CBF and pain intensity ratings. (B) A plot of the group mean tonic pain ratings vs the absolute CBF in the contralateral dpIns. (C) Group mean absolute CBF (black) extracted from the peak dpIns cluster are plotted alongside the ongoing pain intensity ratings (gray) over time. Error bars represent the SE of the mean (from Ref. 77). ASL, arterial spin labeling; CBF, cerebral blood flow.

A key factor highlighted by the Owen et al. 2012 study^66^ that may explain the lack of a strong coupling between the rCBF and the psychophysics data was the pulsed ASL method used to capture these relationships. By comparison, pcASL approaches boost SNR by ∼30% compared with pASL.^19^ Another way of further optimizing the ASL protocol would be to incorporate multiple delay times (multidelay pcASL). This method would be particularly helpful in paradigms aimed at elucidating how perfusion is dynamically changing over a slowly fluctuating pain experience, because it benefits from capturing slow (ie, over many minutes) rather than fast (ie, over a few TRs) changes. A study by Segerdahl et al.^77^ demonstrated the utility of this approach, interrogating rCBF changes triggered by an experimental medicine model of capsaicin-induced heat hyperalgesia using a multi-PLD ASL approach (Fig. 4). When applied to the skin, capsaicin triggers a complex pain experience that entails cutaneous pain sensations that can last for hours. Topical and intradermal capsaicin administration has been studied previously with BOLD fMRI, identifying brainstem and supraspinal features likely to contribute to central sensitization induced secondary hyperalgesia in response to acute mechanical stimulation.^7,45,57,102^ In the Segerdahl study, over a 2-hour multidelay ASL session, the topical capsaicin was instead used for its capacity to elicit ongoing pain in the primary area of application. Therefore, it was possible to explicitly model both fast and slow pain dynamics (ie, the slow onset and gradual habituation of the topical capsaicin in addition to the rapid onset of both heat hyperalgesia and cooling-induced relief in the primary zone). Alongside identifying several brain regions active during the onset of pain and its maintenance, key results from this study confirmed that the insula is activated by different dynamic features of the ongoing pain state: the caudal anterior and dorsal posterior insula were both active during the peak of the capsaicin-induced pain state, while only the dorsal posterior insula contralateral to the site of capsaicin administration tracked the dynamic changes in nociceptive input and subjective intensity over the full paradigm. These findings also confirmed, without the use of any ionizing radiation, those that Iadarola et al.^36^ obtained using ^15^O water PET imaging of multiple phases of pain evoked by intradermal capsaicin injection, particularly during the later, waning period.

Overall, by harnessing the strengths of ASL, it has been possible to uniquely identify core neurobiological features linked to different features of experimentally induced tonic and ongoing pain states. The next step is to translate these findings into new experimental approaches—for example, computational,^26^ intracortical electrophysiology,^50–52^ and experimental brain stimulation approaches^48^—that are better suited to interrogate the relationships between elements of pain such as saliency, nociception, and subjective experience that are essential for the development of novel efficacious pain-relief strategies.

## 5. Imaging postsurgical pain with arterial spin labeling

In light of the suitability of ASL to examining tonic states, investigation of patients experiencing spontaneous pain (a defining characteristic of chronic pain) is a clear avenue for study. Spontaneous pain is ever-present and typically cannot be “switched off” under experimental control. Healthy volunteer investigations of induced ongoing pain after a planned surgical intervention provide a well-standardized environment for study. Using within-subject “cross-over” designs, the effects of interindividual differences in pain history, distribution, psychological, and genetic factors are minimized as each participant acts as their own experimental control.^41^ Investigations of induced tonic pain after third molar (wisdom tooth) extraction (TME) have demonstrated important proofs-of-concept of the utility of ASL in understanding the cerebral representation of pain and treatment response.

The TME model reliably produces moderate-to-severe ongoing pain.^6^ It is commonly performed in healthy young adults who unlike chronic pain patients can be investigated free of pain and its associated sequelae^59^ and provides the additional benefit of meeting the design requirements of cross-over analgesic trials. Importantly, unlike other experimentally induced models, the experience is due to trauma to soft tissue and bone, resulting in prolonged and inescapable pain; qualities likely to engage endogenous pain control systems.^23^ Accordingly, the acute inflammatory response associated with the surgical extraction has rendered the TME model a gold standard for assessing analgesics.^6^

**Figure 5.**
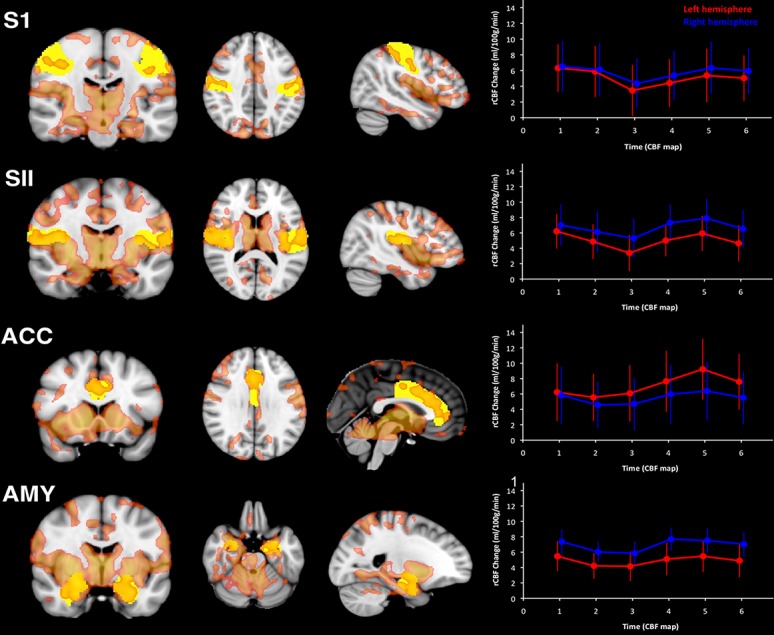
Imaging postsurgical pain with ASL. Elevations in rCBF immediately after third molar tooth extraction. Voxels in red designate significant increases in rCBF postsurgery. A priori defined regions of interest (ROIs) for key anatomical areas of the brain are in yellow. Line plots indicate the magnitude of the increase in rCBF during postsurgical pain, compared with pain-free presurgical periods in each ROI in left hemisphere (red line) and right hemisphere (blue line). Each data point represents the mean rCBF increase (in millilitres/100 g/min) during each individual pCASL scan. ACC, anterior cingulate cortex; AMY, amygdala; S1, primary somatosensory cortex; SII, secondary somatosensory cortex (from Ref. 34). ASL, arterial spin labeling; pCASL, pseudocontinuous ASL; rCBF, regional cerebral blood flow.

Pseudocontinuous ASL investigation of post-TME pain demonstrated reliable rCBF increases, of the order of 5% to 10%, in brain regions known to underpin the pain experience including thalamus, primary and secondary somatosensory, and posterior and anterior insular cortices^34^ (Fig. 5). Regional cerebral blood flow increases were largely stable within an experimental session and related to patients' subjectively reported pain in many of these areas. Examination of within-session test–retest reliability using intraclass correlation coefficients indicated that repeated pCASL assessments are extremely reliable (intraclass correlation coefficient, ICC > 0.9),^32^ and that within-subject between-session reliability assessments of pain-free and postsurgical pain states also demonstrate good reliability (ICC ≥ 0.6). In short, reliable rCBF estimates of ongoing pain can be derived using within-subject repeated-measures designs akin to those used in cross-over analgesic trials.

## 6. Imaging chronic pain with arterial spin labeling

**Figure 6.**
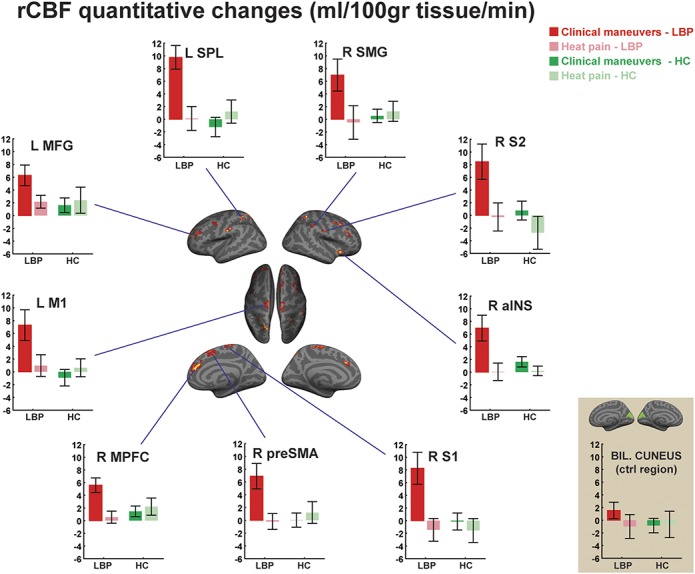
Imaging chronic pain with ASL. In cLBP patients, rCBF elevations were observed after pain-exacerbating clinical maneuvers (red), but not after the resolution of heat pain (pink) or in healthy volunteers receiving the same stimuli (green) (from Ref. 91). ASL, arterial spin labeling; aINS, anterior insula; BIL, bilateral; cLBP, chronic low-back pain; CTRL, control; HC, healthy controls; L, left side; M1, primary motor cortex; MFG, middle frontal gyrus; MPFC, medial prefrontal cortex; preSMA, presupplementary motor area; R, right side; rCBF, regional cerebral blood flow; S1, primary somatosensory cortex; S2, secondary somatosensory cortex; SMG, supramarginal gyrus; SPL, superior parietal lobule.

Because of its sensitivity to brain signals evolving over minutes, or longer, ASL is also particularly well-positioned to investigate the brain mechanisms underlying chronic pain. The first experiment applying ASL to the study of a group of chronic pain patients was performed on chronic low-back pain (cLBP) patients by Wasan et al.^91^ During an imaging visit, both cLBP patients and healthy, pain-free volunteers were scanned with pulsed ASL immediately before and after a period of “clinical maneuvers” (such as straight leg raises or pelvic tilts) designed to exacerbate patients' own low-back pain but were not painful for the controls. By the time of the second ASL scan following the clinical maneuvers, patients reported a clinically relevant (>30%) increase in pain. Pain exacerbation in the patients was accompanied by an rCBF increase (of 6–10 mL/100 g of tissue/min) in regions including S1, S2, the anterior insula, and MPFC in patients after the clinical maneuvers. By contrast, when the same participants were imaged before and after exposure to individually calibrated heat stimuli (as opposed to the maneuvers), pain did not linger after stimulation nor were increases in rCBF observed (Fig. 6). These findings indicated that the maneuver-induced rCBF changes observed were specific to clinical pain exacerbation, as opposed to pain receipt per se or a confounding effect of serial MRI assessments.

Arterial spin labeling examinations of clinical pain have become more popular in recent years. Studies of different painful conditions including inherited erythromelalgia,^79^ osteoarthritis of the hand^35,40^ and knee,^18^ trigeminal neuralgia and temporomandibular disorder,^29,100^ fibromyalgia,^81^ migraine, including pediatric migraine,^15,33,39,101^ interstitial cystitis/chronic pelvic pain,^22,92^ chemotherapy-induced painful neuropathy,^64^ postherpetic neuralgia,^54^ spondylotic myelopathy,^106^ painful diabetic polyneuropathy (DPN),^78^ rheumatoid arthritis,^46^ Gulf War Illness,^49^ and complex regional pain syndrome^80^ have been reported.

Studies are also beginning to demonstrate the power of integrating ASL data with other modalities to identify novel features of different chronic pain conditions. For example, a study by Segerdahl et al.^78^ used a multimodal clinical neuroimaging approach to test whether the sensory phenotype of painful DPN is linked to altered function of the ventrolateral periaqueductal gray (vlPAG)—a key node of the DPMS. Data from this study showed that the vlPAG has significantly altered resting functional connectivity (as observed with BOLD FMRI) in patients suffering from painful DPN, which was positively correlated with these patient's spontaneous pain intensity ratings. In addition, the study showed that the strength of the functional connectivity between the vlPAG and the rostral anterior cingulate—another key node of the DPMS—was positively correlated with the painful DPN patient's allodynic pain and the magnitude of their cortical response elicited by an experimental tonic heat paradigm that was imaged with ASL. No such relationship was observed for the painless DPN patients, however. By combining these different data sets, the study was able to identify a dysfunctional node in the DPMS, which may be contributing to painful but not painless DPN. Another recent study by Lee et al. used a machine learning approach to build a classifier from a combination of resting-state BOLD FMRI, ASL, and heart rate variability data to predict differences in low back pain severity, both within and between subjects. Data from this study show that the accuracy of prediction (for both within- and between-patient pain endpoints) was highest for rCBF data computed from ASL, than for S1 connectivity (S1_CONN_) or high-frequency heart rate variability (HF_HRV_). Furthermore, combining multimodal parameters (rCBF + S1_CONN_ + HF_HRV_) produced the best classification performance compared with using each modality in isolation.^44^ Overall, these studies provide examples of how the richer dimensionality obtained by adding ASL to an imaging session can inform our understanding of brain mechanisms of pain, and/or aid in the identification of imaging markers of pain.

Although previous studies required some kind of stimulation/exacerbation for changes in rCBF to be apparent, ASL facilitates resting-state examination of brain activity underpinning spontaneous pain in the absence of any manipulation. Using pCASL, Howard et al.^35^ were able to evaluate the rCBF correlates of hand pain due to osteoarthritis of the carpometacarpal joint of the thumb. They demonstrated elevated resting rCBF in widespread regions including the core regions of the default mode network (medial prefrontal cortex [MPFC], posterior cingulate cortex, hippocampal formation, lateral temporal cortex, and inferior parietal lobule) as well as the primary (hand representation) and secondary somatosensory cortices, anterior cingulate cortex, dorsolateral prefrontal cortex, insula, thalamus, insula, thalamus, PAG/nucleus cuneiformis, and amygdala. By taking advantage of the naturally occurring variation in pain over time, the authors further compared the change in pain with changes in rCBF across 2 separate sessions in the patients, observing that in 2 regions (L amygdala and thalamus), the session-wise increase in pain was accompanied by a proportional increase in rCBF (whereas other regions, such as the S1, demonstrated the opposite relationship).

**Figure 7.**
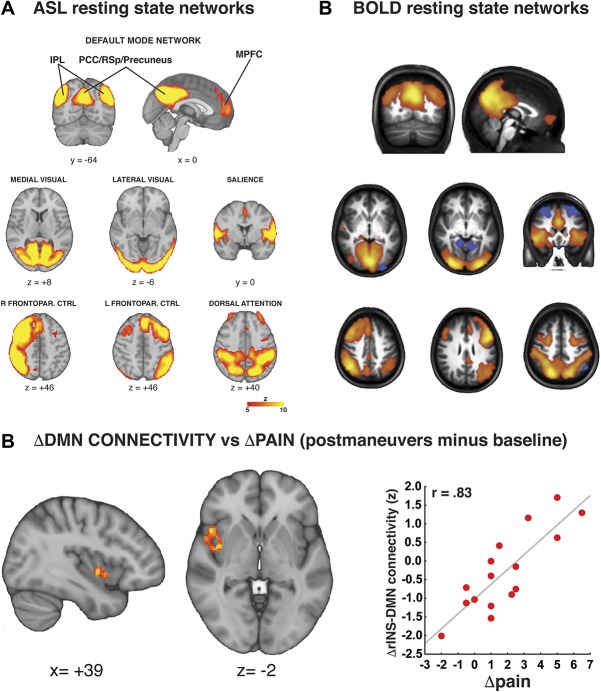
Imaging functional connectivity with ASL. Arterial spin labeling allows for the identification of resting-state networks (A: from Ref. 55) similar to those identified with BOLD fMRI (B: adapted from Ref. 107). (C) Functional connectivity analyses of ASL data reveal increases in DMN-insula connectivity that is proportional to experimentally induced increases in clinical pain in cLBP patients.^55^ ASL, arterial spin labeling; BOLD, blood-oxygen level–dependent; DMN, default mode network; fMRI, functional magnetic resonance; pgACC, pregenual anterior cingulate cortex; IPL, inferior parietal lobule; LTC, lateral temporal cortex; MPFC, medial prefrontal cortex; PCC, posterior cingulate cortex; RSp, retrosplenial cortex; Frontopar. CTRL, frontoparietal control.

In addition to deriving measurements of rCBF, several studies have begun exploring the use of ASL as a tool to evaluate functional connectivity,^16,54,55,82^ further underscoring the remarkable flexibility of this technique, and the impressive richness of the data set that it can generate. By performing a dual regression–independent component analysis of perfusion-weighted time series, Loggia et al.^55^ were able to show that several of the “canonical” resting-state networks can be identified from ASL data (Fig. 7). Using the same analytical methods, these authors went on to show that the severity of clinical pain in cLBP patients was correlated with the strength of functional connectivity between the middle insula and the default mode network. This relationship was confirmed both at baseline (ie, spontaneously reported pain before any experimental manipulation correlated with DMN-insula connectivity) and after the above-mentioned pain-exacerbating clinical maneuvers (ie, pain increases induced by the maneuvers correlated with increases in DMN-insula connectivity). Intriguingly, a seminal paper by Napadow et al.^62^ had previously demonstrated, using BOLD fMRI, that DMN-insula connectivity was also correlated with the severity of spontaneous clinical pain in fibromyalgia. In fact, similar BOLD-based results have subsequently been reported for other conditions such as osteoarthritis and complex regional pain syndromes, and further confirmed in cLBP patients.^5^ In these cases, the severity of clinical pain was found to correlate with the strength of connectivity between the insula and the MPFC (a core region of the DMN^8^).

Although the use of functional connectivity on ASL is still very limited, at least one other study has applied this approach to the use of a chronic pain condition. Liu et al.,^54^ used ASL to evaluate both alterations in both perfusion and connectivity in patients with postherpetic neuralgia. They observed rCBF elevations in various regions, including S1, insula, basal ganglia, inferior parietal lobule (in some cases correlating with pain severity), and decreases, mostly located in DMN regions. Seed-based functional connectivity analyses of the same data set revealed elevated connectivity between the left caudate nucleus and a widespread set of regions, including thalamus, posterior cingulate cortex, basal ganglia, insula, MPFC, parahippocampal gyrus, superior parietal lobule, and the lateral temporal cortex. The observation that a common neuroimaging metric—albeit varying anatomically—that measures coupling changes to the DMN seems to encode clinical pain in different patient populations raises the intriguing possibility that such measures may reflect a general feature of chronic pain.

Although the feasibility of performing functional connectivity analyses using has been demonstrated, it should be pointed out that BOLD fMRI data still largely dominates the field of functional connectivity, and it is still considered the gold standard because of higher spatiotemporal resolution. In particular, because ASL typically has—as previously mentioned—an “effective TR” that is significantly longer compared with that of BOLD fMRI, the number of time points it can sample for a given scan duration is reduced, which ultimately limits its statistical power to detect functional connectivity patterns. This limitation can be compensated with longer scan durations, although at an increased risk of participant discomfort and/or data quality degradation (due to the higher likelihood of the participant falling asleep, moving, etc). Overall, the tighter coupling between ASL-derived rCBF measurements and neuronal activity, as compared to BOLD,^43,96^ makes the use of perfusion data an attractive method to estimate resting-state networks.^14^

## 7. Imaging response to interventions with arterial spin labeling

Arterial spin labeling, whether in conjunction with some kind of pain stimulation or not, can be used as a tool to track treatment response in the context of nonpharmacological or pharmacological interventions. For instance, Zeidan et al. pioneered the use of ASL in meditation studies.^103–105^ One of these studies^104^ showed that brief (4 days) mindfulness meditation training induced reductions in heat pain–related activation of the primary somatosensory cortex contralateral to the stimulated leg. The authors also observed changes in pain-related activation in various regions, which were proportional to the meditation-induced reduction in pain intensity (ie, increased activity in the anterior cingulate cortex and anterior insula) or pain unpleasantness (ie, increased activity in the orbitofrontal cortex and decreased activity in the thalamus).

Arterial spin labeling was also used to evaluate the brain correlates underlying the effects of transcranial direct current stimulation (tDCS) on the perceived intensity of the capsaicin-induced tonic pain state.^53^ Anodal (but not sham) tDCS applied to the left dorsal lateral prefrontal cortex (to drive the DPMS) successfully reduced the subjective pain intensity of the topical capsaicin paradigm. Crucially, ongoing activity within the left posterior insula and thalamus, as measured using ASL, showed a concomitant tonic reduction in capsaicin-induced rCBF to this anodal tDCS stimulation. Furthermore, the magnitude of this CBF decrease in the posterior insula was significantly correlated with the magnitude of the decrease in subjective pain intensity across subjects, as well as the magnitude of the fractional anisotropy of white matter integrity between the dorsal lateral prefrontal cortex and anterior thalamus. In effect, these data show how brain structure links to functional and behavioral variability after brain stimulation. In addition, these data confirm that ASL is able to read out key neurophysiological features of tonic pain in addition to being sensitive to the pain-relieving effects of noninvasive neuromodulation by tDCS.

Arterial spin labeling can also be used to image the effects of pharmacological treatments. Among the first studies using this approach was the one performed in 2010 by Kato et al.,^39^ which imaged a single 32-year-old male migraineur. When scanned within 1 hour from the onset of a migraine attack, the patient demonstrated various brain perfusion changes, including the hypoperfusion of the medial thalamus, compared with a scan collected interictally. Interestingly, 30 minutes after abortive treatment with rizatriptan (a 5-TH1 receptor agonist of the triptan class), the patient demonstrated an increase in thalamic rCBF, suggesting that normalization of thalamic perfusion might be a mechanism mediating the migraine-abortive action of the medication.

Another important application of ASL is the ability to determine central effects of acutely administered drugs. At its simplest, this may be a determination of whether drugs cross the blood–brain barrier.^68^ The third molar extraction model, as discussed above, facilitates discrete identification of central effects of drugs relating to, and independently of, their analgesic action. Investigations of the analgesic mechanism of action of ibuprofen, a commonly prescribed nonsteroidal anti-inflammatory drug, compared with placebo, demonstrated no effects on rCBF when participants were pain-free, but that surgery-induced rCBF increases were largely normalized by treatment.^31^ However, post-treatment rCBF increases during postsurgical pain were observed in the periaqueductal gray and rostral ventral medial medulla, suggesting engagement of endogenous descending pain control mechanisms, findings not previously described in humans but in accord with preclinical reports of central effects of NSAIDS in the presence of an ongoing noxious afferent peripheral drive.^47^

Overall, these findings provide a proof-of-concept demonstration of the utility of perfusion MRI-based indices of pain as adjunctive endpoints during the development of novel therapeutics, whether nonpharmacologic or pharmacologic.

## 8. Future directions

As previously mentioned, an essential benefit of ASL fMRI is the fact that it enables quantification of brain activity in absolute physiological units and recent advances in the field aim to improve the sensitivity and robustness of this capacity. In addition, newer ASL sequences are now able to selectively quantify perfusion arising from specific arteries (eg, vessel-encoded ASL; see: Ref. 95) or globally label the blood based on its velocity characteristics (eg, velocity-selective ASL; see: Ref. 97) and are promising research areas to take note of Refs. 30 and 38. The aim of this section is to highlight a few developments that are well suited for translation to a pain neuroimaging setting.

### 8.1. Ultra–high-field Arterial spin labeling

A key focus of ASL sequence development is to improve the SNR of the technique. As discussed previously, ASL has low SNR due to the low proportion of blood signal compared with background signal in cortical tissue. This necessitates long acquisition times, often at a reduced spatial resolution to obtain robust data. Adoption of ASL to an ultra–high-field (UHF) MRI (main magnetic field strength, B_0_ ∼ 7 Tesla or higher) setting will improve upon these inherent weaknesses. Specifically, a core benefit of UHF MRI imaging is the inherent boost in available signal combined with longer blood T1 relaxation. It is estimated that an increase of 300% to 400% SNR are expected at 7T compared with 3T.^85^ Practically, this means that data of similar quality to what is observed at 3T could be collected in a fraction of the scan time; alternatively, this boost in SNR could be used to enhance the spatial resolution of the perfusion images collected within a given scan. Although UHF ASL is still under development and there are a number of technical challenges yet to overcome, it will provide a powerful boost in the sensitivity, speed, and spatial resolution at which rCBF can be quantified and therefore offers considerable promise for a broad range of experimental and clinical pain applications.

### 8.2. Velocity-selective arterial spin labeling

Most ASL approaches currently use an arterial labeling strategy that is “spatially selective,” which means that only arterial blood flowing upstream through a given plane perpendicular to the internal carotid and vertebral arteries is magnetically inverted. The amount of signal that is observed when the images are collected is thus dependent on how much signal is transited to a given voxel before that signal decays—a factor that depends on the spatial distance between the labeling plane and a given voxel and the speed of the blood. A powerful alternative approach called “velocity-selective” ASL is able to label arterial spins based on how fast they are flowing irrespective of their spatial location.^63,72,97,99^ Essentially, velocity-selective ASL makes it possible to label and measure the arterial spins in a voxel without the drawbacks related to transit delays mentioned previously. A number of sequence optimizations are being developed to tackle some of the SNR-related drawbacks to this method, with promising success.^30,71,72^ We anticipate that this method could provide a helpful means of quantifying perfusion in tissue types (eg, white matter) and anatomical locations (eg, the spinal cord) that are otherwise beyond the scope of the spatially selective methods that are currently available.

## 9. Conclusions

Although the application of ASL to the study of pain is still in its infancy, with fewer than 50 scientific peer-reviewed articles published to date, this technique has already demonstrated to be a valuable tool. Its sensitivity to slowly fluctuating brain signals makes it particularly suitable for the evaluation of correlates of persistent pain, including postsurgical and various clinical pain conditions. Because ASL can generate rCBF in absolute, physiological units, it can be fully quantitative, unlike, for example, BOLD fMRI, which typically yields only relative metrics. ASL is also a very versatile tool because it facilitates not only the assessment of simple perfusion changes (the creation of rCBF maps “at rest” or during an experimental condition, akin to ^15^O water PET), but also the evaluation of more complex and dynamic neural changes opening the door to novel paradigm designs and the computation of functional connectivity measures (much like BOLD fMRI). Finally, because ASL does not require the intravenous administration of a radiopharmaceutical or contrast agent, this technique is less-invasive, generally very safe, and thus well suited for within-subject longitudinal designs.

## Disclosures

The authors have no conflict of interest to declare.

M.L. Loggia is supported by DoD-W81XWH-14-1-0543, R01-NS094306-01A1, R01-NS095937-01A1, and 1R01DA047088-01. M.A. Howard is supported by a Medical Research Council Experimental Medicine Challenge Grant award (MR/N026969/1) and the NIHR Biomedical Research Centre for Mental Health at the South London and Maudsley NHS Trust. A.R. Segerdahl and I. Tracey are supported by the Wellcome Trust and Medical Research Council.
